# Association between Oral Microbiome and Gastroesophageal Reflux Severity

**DOI:** 10.3390/jcm13154479

**Published:** 2024-07-31

**Authors:** Declan J. Power, Vincent Ho, Jerry Zhou

**Affiliations:** School of Medicine, Western Sydney University, Campbelltown, NSW 2560, Australia; 17661010@student.westernsydney.edu.au (D.J.P.); v.ho@westernsydney.edu.au (V.H.)

**Keywords:** gastroesophageal reflux diseases, microbiome, acid reflux, oral microbiota, DeMeester score, microorganism

## Abstract

**Background/Objectives:** Gastroesophageal reflux disease (GORD) is caused by gastric contents refluxing back into the oesophagus and oral cavity. It can lead to injuries to the mucosa in the form of erosion and ulcers. Our past research have shown acid reflux severity and disease progression is associated with alternations in the microbiota of the distal oesophagus. The aim of this study was to explore whether changes in the oral microbiota occurred in GORD patients and establish any associations with reflux severity. **Methods:** Fresh mouthwash samples were collected from 58 patients experiencing reflux symptoms referred for 24 h pH monitoring. The participants were categorised into three groups based on their DeMeester scores: Normal (<14.72), Mild (14.2–50), and Moderate/severe (>51). Microorganism identity and diversity were generated using hypervariable tag sequencing and analysing the V1–V3 region of the 16S rRNA gene. **Results:** No differences in microbiota diversity were found in oral microbiota between groups using the Chiao1 diversity index and Shannon diversity index. Microbiota in the Mild group showed reductions in *Rothia dentocariosa* and *Lautropia*, while *Moryella* and Clostridiales_1 were increased compared with the Normal group. In the Moderate/severe group, the abundance of *Rothia aeria* was reduced compared with the Normal group, while *Schwartzia*, Rs_045, *Paludibacter*, *S. satelles*, *Treponema*, and *T. socranskii* all had increased abundance. The abundance of *Prevotella pallens* was higher in the Mild group compared with Moderate/severe, while *S. satelles* and *Paludibacter* abundances were lower. **Conclusions:** Our study shows the oral microbiome show significant differences between acid reflux severity groups, as categorised by DeMeester score.

## 1. Introduction

Gastroesophageal reflux disease (GORD) is a common gastrointestinal (GI) disease, with an estimated prevalence of 18.1–27.8% in North America and 2.5–7.8% in Asia [1]. GORD is characterised by the abnormal reflux of stomach and duodenal contents into the oesophagus and is often accompanied by troublesome symptoms such as heartburn, regurgitation, or complications (e.g., asthma, cough, and laryngitis). Current diagnostic testing for GORD includes symptom questionnaires, response to therapeutic intervention, upper endoscopy, oesophageal manometry, and reflux monitoring. 

Disturbances in the balance of the human GI microbiome are associated with a number of diseases [2]. Our previous study identified distinct microbiota populations in the distal oesophagus that are associated with stages in the gastroesophageal reflux disease spectrum [3]. The identification of these microbes provided insights into the mechanisms of disease progression and potential biomarkers for diagnostic screening. Oesophageal microbiota sampling using upper GI endoscopy is invasive; therefore, we sought to determine whether similar alterations in microbiota are evident in the oral cavity and if a non-invasive oral sampling of patients with acid reflux would produce a similar microbiota alteration associated with acid reflux severity. 

The oral cavity harbours a highly complex microbiome, second only to the colon in complexity. An association between oral microbiome dysbiosis and its impact on human health has been reported in many systemic diseases, including autoimmune diseases, systemic malignancies, and pregnancy outcomes [4]. A recent study found significant changes in microbiota composition in GORD patients compared with healthy controls [5]. A similar study comparing the oral microbiota of patients with reflux esophagitis and healthy controls found differences at the phylum and genus levels [6]. The oral cavity ecosystem is altered during GORD, often through a reduction in salivary pH, mucosal inflammation, and tooth erosion; consequently, the oral microbiota may also be impacted by these factors. Moreover, the migration of gastric microbiota into the oral cavity as a result of refluxate can further alter the oral microbiome in GORD patients. 

We hypothesis that the composition of the oral microbiome changes in concert with changes in acid reflux severity. In this study, we investigated the relationship between oral microbiota and acid reflux severity using 24 h pH monitoring and high-throughput DNA sequencing.

## 2. Materials and Methods

### 2.1. Participants

Participants between the ages of 18–70 years old were enrolled prospectively from the Gastrointestinal Motility Clinic, Camden Hospital (NSW, Australia) from November 2021 to August 2022. All participants were clinically suspected of GORD and presenting with typical GORD symptoms, such as heartburn and regurgitation. Patients were referred to the clinic for routine oesophageal manometry and 24 h ambulatory pH monitoring. Exclusion criteria were the presence of other oesophageal motility disorders (e.g., achalasia, spasm), oesophageal hypersensitivity, neurological disorders, gastrointestinal disorders, oral diseases, infection, malignancy, immunodeficiency, metabolic diseases, or any immunological disorders. Pregnant and nursing women, active smokers, or individuals on antibiotics or probiotics were also excluded. History of acid suppression medication use was not an exclusion criterion, as all participants in this study were medicated with proton pump inhibitor (PPI) for at least 4 weeks prior to taking part in this study, but abstained from PPI use 14 days before pH monitoring.

Given the exploratory nature of this study, a power calculation could not be used to determine the recruitment target. Instead, similar microbiota studies were used as references [3,5,6,7]. A median of 19 participants per group or a total of 58 participants was determined as the recruitment target; whichever was reached first. 

The study was approved by the ethics committees of South Western Sydney Local Health District (Ethics ID: 2019/ETH13045) on 16 November 2019. The procedures were performed in accordance with the Declaration of Helsinki. Informed consent to participate was obtained for all participants. 

### 2.2. Gastroesophageal Reflux Disorder

Participants were categorised into groups based on their DeMeester scores obtained through 24 h pH monitoring and data acquisition software 6.1.1. (Digitrapper pH-Z and AccuView, Medtronic, Minneapolis, MN, USA). The DeMeester score is a composite score of the acid exposure during a prolonged ambulatory pH monitoring to categorise GORD patients. Participants with a DeMeester score [8] of less than 14.72 and less than 4% acid exposure time [9] were considered to be within normal ranges of acid reflux and were classified into the Normal group; patients with scores of 14.72–50 (mild GORD [10]) and ≥4% acid exposure time were classified into the Mild group, and patients with scores greater than 51 [10] (moderate to severe GORD) and ≥4% acid exposure time were classified into the Moderate/severe group. 

### 2.3. Microbiota Sampling and DNA Extraction

Samples were collected at the end of the 24 h pH monitoring study. A diet sheet of foods with pH above 4 and a sample daily menu was provided to all patients, who were asked to attempt to eat similar foods. Patients were instructed to refrain from smoking, drinking alcoholic beverages, tooth brushing or using mouthwash for the duration of the 24 h pH monitoring period until samples were collected. Participants were also asked to fast for 8 h prior to sampling; water was allowed. Sample collection involved participants rinsing their mouths thoroughly for 30 s with 10 mL distilled water, which was collected and immediately frozen and stored at –80 °C until assayed. DNA extraction utilised the PureLink Microbiome DNA Purification Kit (ThermoFisher, Carlsbad, MA, USA). Microbiome 16s rRNA sequencing was performed by Australian Genome Research Facility (SA, Australia). Amplicon sequencing targeting the hypervariable region (V1–V3, 27F/529R) of the 16S rRNA gene was performed on the Illumina MiSeq platform (Illumina, San Diego, CA, USA), using the Illumina Nextera XT Index with paired-end sequencing. Raw paired-end Illumina reads were trimmed using Cutadapt 4.9. Sequence analysis was performed using Quantitative Insights into Microbial Ecology 2 (QIIME Version 8.0.1623). QIIME was also used to generate amplicon sequence variants (ASVs). Sequences were normalised to relative abundance of reads per million. Sequences were then clustered into operational taxonomic units (OTUs) following the default QIIME2 pipeline based on 99% sequence similarity against the Greengenes database, version 13.8. The OTU abundance information was normalised by rarefaction; 29,416 readings were extracted from each sample under conditions of sequencing at a sufficient depth. A heatmap was created from the abundances of top features. Five indices were applied to calculate the alpha diversity, including Observed species, Chao1, Shannon, Simpson, and ACE. QIIME 2 (2024.2) software was used to calculate beta diversity on both weighted and unweighted unifrac values, and differences among samples in species complexity was evaluated by beta diversity analysis. Distance ordination was plotted using the Principal Co-ordinates Analysis (PCoA), distance method Bray–Curtis Index and pairwise PERMANOVA analysis. Spearman’s rank correlation coefficient was calculated to estimate linear correlations between variables. Tests were conducted with linear discriminate analysis (LDA), effect size (LEfSe), and EdgeR.

### 2.4. Statistical Analysis

The Kolmogorov–Smirnov test was applied to verify the normality of the data. Data for continuous variables were expressed as mean ± standard deviation or median interquartile range (IQR), and categorical variables were expressed as numbers (percentages). Student’s *t*-tests were used to compare the averages of continuous variables and Mann–Whitney U tests, and chi-square tests were used to compare the percentages of categorical variables between groups. These statistical analyses were completed using SPSS 19.0 software. Visualisation and statistical analyses of sequencing data were performed using Microbiome Analyst 2.0 [11] (www.microbiomeanalyst.ca accessed 3 May 2024), which includes diversity analysis, EdgeR, LEfSe, and Spearman’s rank correlation analysis. *p* < 0.05 was considered statistically significant.

## 3. Results

A total of 58 suspected GORD patients with reflux symptoms were profiled for this study (Table 1). No participants were edentulous. The median age was 61 years old and the median DeMeester score was 17.5. There was a slight female bias (59%) in the study cohort. Twenty-five (43%) patients reported a DeMeester score within normal limits (<14.72), while 33 (57%) patients reported an abnormal DeMeester score and were diagnosed with GORD. The majority (64%) of GORD patients had mild GORD (DeMeester 14.72–50) while 36% were categorised as having moderate or severe GORD. There were no significant differences in gender, age, and body mass index between study groups. 

Within the study groups, there was a female bias in the Normal (68%) and Mild (57%) reflux groups, while the Moderate/severe group had more males (58%). Similarly, the median ages for the Normal and Mild groups were both 61 years old, while the Moderate/severe group was slightly younger with a median of 53 years old. 

**Figure 1:**
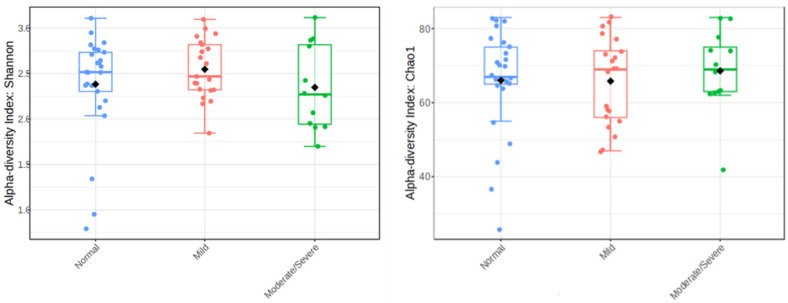
Alpha diversity analysis using Shannon and Chao1 diversity indexes. The colours denote study group: Normal (blue), Mild (red), and Moderate/severe (green).

Comparisons of microbiota diversity between study groups were conducted using Shannon and Chao1 diversity measures (Figure 1). The Shannon scores for the Normal group was 2.5, the Mild group was 2.5, and the Moderate/severe was 2.25. The Chao1 index for the Normal group was 67, the Mild group was 69, and the Moderate/severe was 69. No significant differences in alpha diversity were observed between study groups. In addition, Beta diversity analysis did not show differences between the groups. 

**Figure 2:**
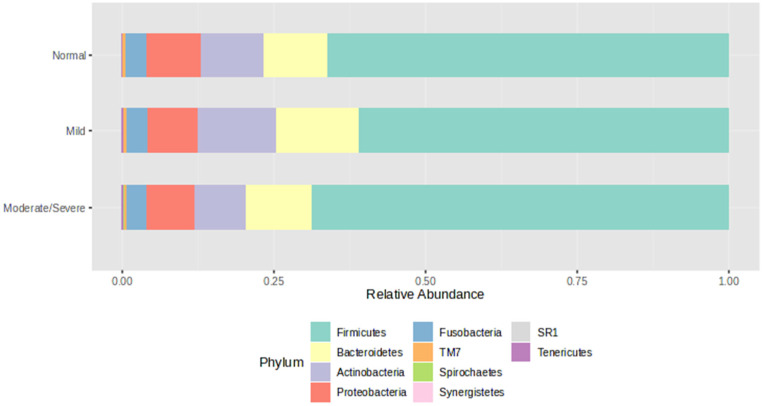
Microbiota phylum composition within each study group.

Relative abundance (%) of microbiota phyla between study groups is shown in Figure 2. Firmicutes was the most abundant phylum in all groups (Normal 66.1%, Mild 61.0%, Moderate/severe 68.8%), followed by Bacteroidetes (Normal 10.6%, Mild 13.5%, Moderate/severe 10.9%), Actinobacteria (Normal 10.5%, Mild 13.2%, Moderate/severe 8.5%) and Proteobacteria (Normal 8.8%, Mild 8.1%, Moderate/severe 7.8%). 

**Figure 3:**
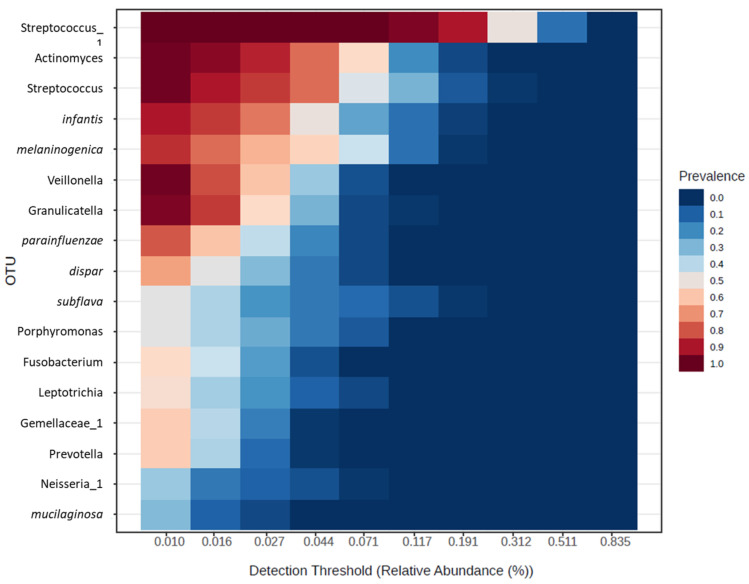
Core oral microbiome ranked by abundance from all samples (n = 58). The sample prevalence if set at 20% and relative abundance threshold at 0.01%.

The core microbiome analysis identified the set of taxa that are detected in a high fraction of the population above a given abundance threshold (Figure 3). Ranking of core OTUs within all samples by relative abundance (%) showed that the genera *Streptococcus* and *Actinomyces* and species *B. infantis* and *P. melaninogenica* were the dominant components of the oral microbiota. 

To investigate the differential abundance between groups at the phylum, family, class, order, genus, and species levels, we used EdgeR pairwise analysis (Table 2). *p* < 0.01 and FDR < 0.05 were determined to indicate statistical significance. *Rothia* species *R. dentocariosa* and *R. aeria*, and Proteobacteria *Lautropia* were more abundant in the Normal group compared with the Mild and Moderate/severe reflux groups. In contrast, species of Firmicutes (*L. moryella*, *S. satelles*), Bacteroidetes (*Paludibacter*), and Spirochaetes (*T. socrankii*) were more abundant in the Mild and Moderate/severe reflux groups than in the normal group. 

Within the reflux groups, *Prevotella pallens* was more abundant in the Mild reflux group compared to the Moderate/severe group; conversely, *S. satelles* and *Paludibacter* were more abundant in the Moderate/severe group than in the Mild group. 

**Figure 4:**
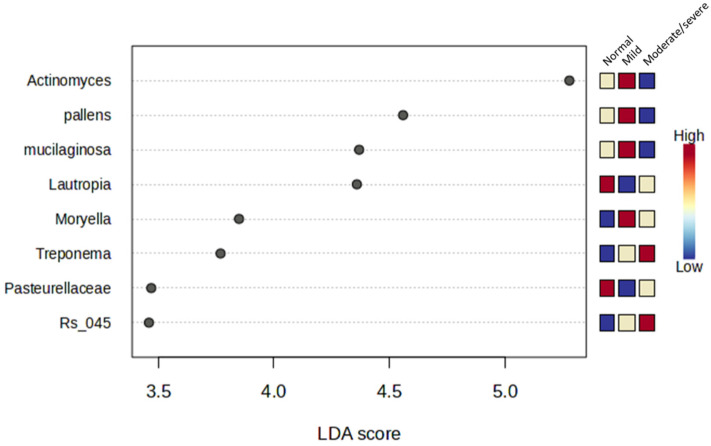
Bacterial taxa diversities between DeMeester groups (Normal, Mild reflux, and Moderate/severe reflux). Bacterial taxa were detected by LEfSe (*p* < 0.05, linear discriminant analysis (LDA) > 2 logs). The dots denote the log Linear Discriminant Analysis (LDA) score. Taxas above log LDA score cut off > 2.0.

To assess the association of different taxa between the reflux groups, LEfSe analysis with LDA score >2 and *p* < 0.05 was performed (Figure 4). Our results identified eight taxa with significantly different abundance between the group: *P. pallens* (*p* = 0.014, LDA = 4.56), Rs 045 (*p* = 0.015, LDA = 3.46), *Lautropia* (*p* = 0.017, LDA = 4.36), *Actinomyces* (*p* = 0.034, LDA = 5.28), *Moryella* (*p* = 0.039, LDA = 3.85), *Pasteurellacea* (*p* = 0.041, LDA = 3.47), *Treponema* (*p* = 0.047, LDA = 3.77), and *mucilaginosa* (*p* = 0.049, LDA = 4.37). 

**Figure 5:**
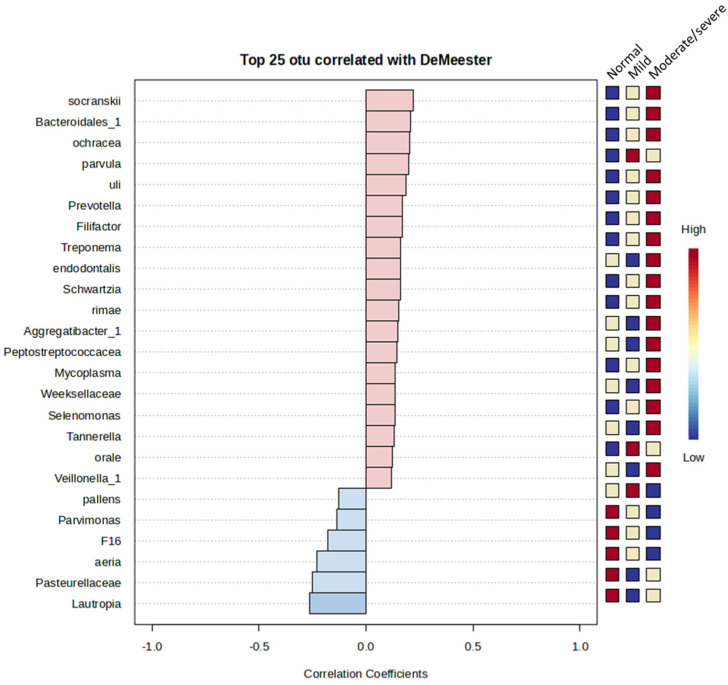
Pattern search plot showing correlation of features correlated with the DeMeester score. The features are ranked by their correlation. Blue bars represent negative correlation while red bars represent positive correlation, and white bars represent no correlation. The deeper the colour (darker blue or red), the stronger the correlation. To the right is a mini heat map showing whether the abundance of that feature is higher (red) or lower (blue) in each group.

Spearman’s rank correlation analysis was performed between microbiota relative abundance and the DeMeester score. The top 25 OTUs that correlate with DeMeester score are shown as a pattern search plot (Figure 5). Although several features correlated with DeMeester score, no statistically significant features were identified. 

The taxa that were positively correlated with DeMeester score severity were *socranskii*, Bacteroidales_1, *ochracea*, *parvula*, *uli*, *Prevotalla*, *Filifactor*, *Treponema*, *endodontalis*, *Schwartia*, *rimae*, Aggregatibacter_1, *Peptrostreptococcacea*, *Mycoplasma*, *Weeksellaceae*, *Selenomonas*, *Tannerella*, *orale*, and *Veillonella_1*. Taxa that showed a negative correlation with DeMeester score severity were *pallens*, *Parvimonas*, *F16*, *aeria*, *Pasteurellaceae*, and *Lautropia*. 

## 4. Discussion

The balance of micro-organisms in the human gastrointestinal tract plays a pivotal role in health and disease. Our previous research had shown that distinct changes in the lower oesophageal microbiota are associated with disease states within the gastroesophageal reflux disease spectrum [3]. In this study, we explore whether similar microbiota changes are seen in the oral cavity of patients with GORD. 

GORD is a condition that is believed to occur due to dysfunction of the lower oesophageal sphincter, which is responsible for preventing the back flow of stomach contents into the oesophagus [12]. This regurgitation of gastric juices into the oesophagus, oropharyngeal and oral cavity lowers their pH [13]. Therefore, exposure to gastric contents in the upper GI tract can cause inflammation of the oral mucosa and loss of tooth structure through enamel erosion. These changes in the oral ecosystem may in turn lead to microbiota alterations.

Previous studies in the oral microbiota of GORD patients found there were no changes in overall diversity between GORD and healthy controls, but several alterations in specific taxa were noted. These changes could be a result of acid reflux affecting the oral ecosystem [5,6]. In our study cohort, we also did not observe significant changes in microbial diversity as measured by the Chao1 and Shannon diversity indices. This is consistent with our previous study findings in the distal oesophagus, where no differences in microbial diversity were observed between patients with reflux oesophagitis, Barrett’s oesophagus, oesophageal adenocarcinoma, and healthy controls [3]. 

The oral bacterial community is dominated by six major phyla, Firmicutes, Bacteroidetes, Proteobacteria, Actinobacteria, Spirochaetes, and Fusobacteria, which account for 94% of the taxa detected (Human Oral Microbiome Database http://www.homd.org accessed on 12 May 2024). We observed the same dominant phyla in the oral microbiota of our study cohort. Differential analysis revealed a higher abundance of Spirochaetes in the Moderate/severe group compared with levels in both the Normal and Mild groups. Spirchaetes have been documented in the healthy human oral cavity but remains one of the less exploited phyla of the oral microbiome, where more than 70% species are yet to be cultured. Elevated levels of Spirochaetes were previously reported in the oral microbiota of GORD patients compared to healthy controls [6]. Another study found that increased Spirochaete abundance correlated with Barrett’s oesophagus and reflux oesophagitis from bacterial populations isolated from distal oesophageal biopsies [14]. An increased abundance of oral Spirochaetes was also observed in the initiation and progression of periodontitis [15,16,17], suggesting their contribution to oral inflammation.

The major constituents of the core microbiome of oral cavity includes *Actinomyces*, *Atopobium*, *Corynebacterium*, *Rothia of Actinobacteria; Bergeyella*, *Capnocytophaga*, *Prevotella of Bacteroidetes*; *Granulicatella*, *Streptococcus*, and *Veillonella* of *Firmicutes*; *Campylobacter*, *Cardiobacterium*, *Haemophilus*, *Neisseria* of *Proteobacteria*; TM7, and *Fusobacteria* [18]. Similar microbiota constituents were also observed in the core microbiome of our study participants, with Gram-positive early colonisers *Streptococcus* and *Actinomyces* being the most abundant. 

Within the Moderate/severe group, the Spirochaete genus *Treponema* and the species *Treponema socranskii* were elevated compared to the Normal group. These bacteria play significant roles in periodontal disease, expressing a wealth of virulence factors that facilitate tissue penetration, destruction, and evasion of host immune responses [17]. Additionally, the Moderate/severe group showed increased abundance of family Rs-045 (Candidatus Saccharibacteria phylum), genera *Schwartzia*, *Paludibacter*, and species *Shuttleworthia satelles* were compared to the Normal group. *Paludibacter* and *Shuttleworthia satelles* were also higher in the Moderate/severe group than in the Mild group. These taxa have been associated with oral health and disease: a reduction in oral Rs-045 abundance is observed in the initial stages of periodontitis [19], while the oral *Paludibacter* population is enriched in early-stage intramucosal oesophageal squamous carcinoma [20]. *Shuttleworthia satelles* was isolated from normal human oral flora [21] 

In the Mild reflux group, the relative abundance of *Moryella* was lower compared with the Normal group, while *Prevotella pallens* levels in the Mild reflux group were significantly higher when compared with the Moderate/severe reflux group. Similar changes in these taxa were noted in the distal oesophagus from our previous study [3], where *Moryella* was shown to be reduced in patients with non-erosive reflux disease compared with GORD-free controls, while *Prevotella pallens* was significantly higher in non-erosive reflux disease compared to its levels in reflux oesophagitis and Barrett’s oesophagus. *Moryella* [22] and *P. pallens* [23] are common commensal bacteria in the oral cavity. In other studies, *P. pallens* has been found to be reduced in the saliva of GORD patients not treated with PPI compared to PPI-medicated patients and healthy controls. It is assumed the lower pH in the oral cavity may be ideal for *P. pallens*, given that no significant differences were found between the saliva of medicated reflux patients and normal controls [7]. The *Actinomyces* genus was found to be more abundant in the Mild group compared with the Normal, consistent with reports in a similar study comparing the salivary bacteria in GORD and GORD-free individuals [24]. Interestingly, *Actinomyces* were lower as reflux patients progressed into the Moderate/severe group. *Actinomyces* are common oral bacteria and several species are associated with the early development of plaque on tooth surfaces [25]. Tooth erosion is seen in 24% of patients with GORD, correlating with reflux severity [26]. We hypothesise that, in individuals with moderate to severe reflux, the prevalence of tooth erosion may contribute towards a reduction in oral *Actinomyces*. 

*Rothia* species *dentocariosa* and *aeria* were shown to be reduced in Mild and Moderate/severe reflux groups compared to the Normal group. This is consistent with findings by Qian et al., where *Rothia* was reduced in GORD compared with healthy controls and negatively correlated with DeMeester score [5]. Furthermore, the altered *Rothia* spp. in GORD patients are shown to be restored following PPI treatment [27], suggesting *Rothia* spp. may be highly sensitive to pH-related changes. We observed a reduction in the genus *Lautropia* in the Mild group compared with the Normal group. A similar reduction in this genus was observed in the saliva of oesophageal squamous cell cancer patients [28] and in patients with periodontitis [29,30]. The successful treatment of periodontitis resulted in a subsequent increase in *Lautropia* abundance [31]. This genus may represent a keystone bacterial group in the mouth; its loss may lead to the proliferation of other proinflammatory bacteria. 

The prevalence of PPI use has led to greater research into its impact on the oral microbiota. PPIs are commonly used to relieve symptoms in patients with GORD by inhibiting gastric acid secretion. A study by Mishiro et al. [32] showed that 4 weeks of PPI usage in healthy individuals was sufficient to significantly alter individual oral microbiota taxa and the overall microbiome diversity compared to before PPI administration. Kawar et al. [7] compared GORD patients treated with PPI with untreated GORD patients and healthy controls. It was reported that PPI-treated GORD had altered oral microbiota compared to untreated GORD patients, but no differences in microbiota taxa were observed between PPI-treated GORD and healthy controls [7]. The study suggests PPI may provide some benefits to restoring the oral microbiota of GORD patients. Our study demonstrates that within PPI-treated patients, distinct oral microbiota associated with acid reflux severity can be observed. A reduction in *Prevotella pallens*, reported between untreated GORD patients and healthy controls [7], is also observed between our Mild group and Moderate/severe group. Given that PPIs have become a mainstay first-line therapy for suspected acid reflux disorders, it could be argued that individuals on PPIs are more representative of the real-world oral microbiome of GORD patients. Furthermore, our participants are symptomatic despite PPI treatment, suggesting a limited response to acid suppression and the ongoing acidification of the upper GI tract. 

Our findings suggest that excessive reflux of gastric acid into the oesophagus can directly or indirectly lead to changes in oral microbiota homeostasis. These distinct microbiota changes may provide insight into the impact of GORD to oral health, acting as an easily accessible marker for GORD severity, levels of oral acidification, mucosal inflammation, and tooth erosion. However, a limitation of our study design is that the pH of oral saliva was not measured directly. It is assumed that GORD patients will have an altered oral ecosystem as a direct result of gastric refluxate and alternation in saliva production. A reduction in saliva pH in GORD patients has been reported in several studies [33], along with lower salivary flow rate and reduced buffering capacity [34]. In addition, we did not measure non-acidic refluxates, such as gastric enzymes and bile salts, which can also affect microbiota populations. A diet guideline was recommended as per pH monitoring protocol but their adhesion to the diet was not monitored. All of our participants were on PPI therapy for at least 4 weeks, but the total duration of their medication use was not recorded. Although alterations in oral microbiota resulting from PPI use are established, the long-term effects of such alterations have yet to be determined. 

## 5. Conclusions

In conclusion, the present study identified specific changes in the oral microbiota associated with increased reflux severity determined by DeMeester score. In comparison to the normal DeMeester group, patients with mild reflux had altered levels of *Rothia dentocariosa*, *Lautropia*, *Moryella* and Clostridiales_1, while patients with moderate/severe reflux has altered levels of *Schwartzia*, *Rs_045*, *Paludibacter*, *S. satelles*, *Treponema*, and *T. socranskii*. The oral microbiota has potential to be a non-invasive biomarker for the severity and oral health of individuals with GORD. 

## Figures and Tables

**Table 1:** Participant characteristics.

Characteristics	Normal Group (*n* = 25)	Mild Group (*n* = 21)	Moderate/Severe Group (*n* = 12)	*p* Value
Gender				
Female, n (%)	17 (68%)	12 (57%)	5 (42%)	0.72
Male, n (%)	8 (32%)	9 (43%)	7 (58%)	0.54
Age, median (IQR)	61 (47–68)	61 (47–64)	53 (40–64)	0.34
BMI median (IQR)	23 (23–26)	24 (23–25)	23 (23–25)	0.91
DeMeester Score, median (IQR)	4.4 (3.2–9.8)	23.3 (18.2–27.2)	96.2 (78.9–142.5)	0.32×10^−5^

Body mass index (BMI); Interquartile range (IQR); Group category: Normal, DeMeester <14.72; Mild, DeMeester 14.72–50; Moderate/severe, DeMeester >51.

**Table 2:** Differential bacteria between reflux severity groups (EdgeR).

**Normal vs. Mild**					
**Phylum**	Taxon Name	log2FC	logCPM	*p* Value	FDR
Actinobacteria	s_dentocariosa	3.22	12.63	3.31 × 10^−5^	0.003
Firmicutes	g_moryella	−1.70	11.50	8.64 × 10^−4^	0.025
Firmicutes	o_Clostridiales_1	−1.76	10.37	8.69 ×10^−4^	0.025
Proteobacteria	g_lautropia	2.15	11.94	0.0011	0.025
**Normal vs. Moderate/Severe**					
**Phylum**	Feature	log2FC	logCPM	*p* Value	FDR
Firmicutes	g_schwartzia	−2.46	7.51	1.50 × 10^−5^	0.001
TM7	f_Rs_045	−2.06	7.95	1.31 × 10^−4^	0.006
Bacteroidetes	g_paludibacter	−2.23	7.98	2.13 × 10^−4^	0.006
Firmicutes	s_satelles	−2.16	7.95	3.06 × 10^−4^	0.007
Bacteroidetes	o_Bacteroidales_1	−1.93	9.21	5.09 × 10^−4^	0.010
Spirochaete		−1.69	9.41	9.03 × 10^−4^	0.010
Spirochaete	g_Treponema	−1.54	7.22	9.83 × 10^−4^	0.015
Spirochaete	s_socranskii	−1.54	7.22	0.002	0.025
Actinobacteria	s_aeria	2.67	11.61	0.003	0.033
**Mild vs. Moderate/Severe**					
**Phylum**	Feature	log2FC	logCPM	*p* Value	FDR
Bacteroidetes	s_pallens	3.36	12.71	2.85 × 10^−4^	0.025
Firmicutes	s_ satelles	−1.94	6.89	6.60 × 10^−4^	0.029
Bacteroidetes	g_Paludibacter	−2.02	7.98	0.001	0.038

Log fold-change (logFC); log counts per million (LogCPM); significant *p* value < 0.01; significant FDR < 0.05.

## Data Availability

The datasets used and/or analysed during the current study are available from the corresponding author upon reasonable request.
